# Supplementary material for: The Systems Biology Research Tool: evolvable open-source software
Source: BMC Syst Biol. 2008 Jun 29;2:55. doi: 10.1186/1752-0509-2-55 (PMC2446383; doi:10.1186/1752-0509-2-55)
Supplement: Additional file 1 — SBRT Archive. An archive of the current version of the Systems Biology Research Tool. [file 1752-0509-2-55-S1.zip › sbrt-1.4.0/doc/developers_guide/api/sbrt/kernel/math/class-use/LinearComb.Term.html]

Uses of Interface sbrt.kernel.math.LinearComb.Term


|  |  |  |
| --- | --- | --- |
| **Overview** \| **Package** \| **Class** \| **Use** \| **Tree** \| **Deprecated** \| **Index** \| **Help** | |  |
| PREV   NEXT | **FRAMES**    **NO FRAMES**     **All Classes** |  |


---


## **Uses of Interface sbrt.kernel.math.LinearComb.Term**

| Packages that use LinearComb.Term | |
| --- | --- |
| **sbrt.kernel.math** | Provides classes and interfaces for creating objects relevant to mathematics. |
| **sbrt.shell.text.math** | Provides classes and interfaces for defining formats for mathematical objects. |

Uses of LinearComb.Term in sbrt.kernel.math

| Classes in sbrt.kernel.math that implement LinearComb.Term | |
| --- | --- |
| `static class` | `AbstractLinearComb.Term<V>`             This class is a skeleton implementation of `LinearComb.Term`. |
| `static class` | `HashLinearComb.Term<V>`             This class is a concrete implementation of `LinearComb.Term`. |

| Methods in sbrt.kernel.math that return LinearComb.Term | |
| --- | --- |
| `LinearComb.Term<V>[]` | `LinearComb.getTermArray()`             Returns the terms of this linear combination as an array. |

| Methods in sbrt.kernel.math that return types with arguments of type LinearComb.Term | |
| --- | --- |
| `java.util.List<LinearComb.Term<V>>` | `LinearComb.getTermList()`             Returns the terms of this linear combination as list. |
| `java.util.List<LinearComb.Term<V>>` | `AbstractLinearComb.getTermList()`             Returns the terms of this linear combination as list. |
| `java.util.Iterator<LinearComb.Term<V>>` | `LinearComb.iterator()`             Returns an iterator over the terms of this linear combination. |
| `java.util.Iterator<LinearComb.Term<V>>` | `HashLinearComb.iterator()`             Returns an iterator over the terms of this linear combination. |
| `java.util.Iterator<LinearComb.Term<V>>` | `AbstractLinearComb.iterator()`             Returns an iterator over the terms of this linear combination. |

Methods in sbrt.kernel.math with parameters of type LinearComb.Term

`static

<V> HashLinearComb<V>`

`HashLinearComb.singleTerm(LinearComb.Term<V> term)`
  
          Returns a single-term linear combination with the provided term.

Constructors in sbrt.kernel.math with parameters of type LinearComb.Term

`HashLinearComb(LinearComb.Term<? extends V>[] terms,
double constant,
double zeroCutoff)`
  
          Constructs a new linear combination from the provided terms and
constant.

Constructor parameters in sbrt.kernel.math with type arguments of type LinearComb.Term

`HashLinearComb(java.util.Collection<? extends LinearComb.Term<? extends V>> terms,
double constant,
double zeroCutoff)`
  
          Constructs a new linear combination from the provided terms and
constant.

Uses of LinearComb.Term in sbrt.shell.text.math

| Methods in sbrt.shell.text.math that return LinearComb.Term | |
| --- | --- |
| `LinearComb.Term<V>` | `LinCombTermFormatV2.parse(java.lang.String term)`             Parses the provided string and returns its corresponding term. |
| `LinearComb.Term<V>` | `LinCombTermFormatV1.parse(java.lang.String term)`             Parses the provided string and returns its corresponding term. |
| `LinearComb.Term<V>` | `LinearCombTermFormat.parse(java.lang.String term)`             Parses the provided string and returns its corresponding term. |
| `LinearComb.Term<V>` | `LinCombTermFormatV2.parse(java.lang.String coefficient, java.lang.String variable)`             Parses the provided strings and returns their corresponding term. |
| `LinearComb.Term<V>` | `LinCombTermFormatV1.parse(java.lang.String coefficient, java.lang.String variable)`             Parses the provided strings and returns their corresponding term. |

| Methods in sbrt.shell.text.math with parameters of type LinearComb.Term | |
| --- | --- |
| `java.lang.String` | `LinCombTermFormatV2.format(LinearComb.Term<? extends V> term)`             Returns a formatted string representation of the provided term. |
| `java.lang.String` | `LinCombTermFormatV1.format(LinearComb.Term<? extends V> term)`             Returns a formatted string representation of the provided term. |
| `java.lang.String` | `LinearCombTermFormat.format(LinearComb.Term<? extends V> term)`             Returns a formatted string representation of the provided term. |

---


|  |  |  |
| --- | --- | --- |
| **Overview** \| **Package** \| **Class** \| **Use** \| **Tree** \| **Deprecated** \| **Index** \| **Help** | |  |
| PREV   NEXT | **FRAMES**    **NO FRAMES**     **All Classes** |  |


---
